# Exploratory attitude survey of homeless persons regarding telecare services in shelters providing mid- and long-term accommodation: The importance of trust

**DOI:** 10.1371/journal.pone.0261145

**Published:** 2022-01-06

**Authors:** Zsuzsa Győrffy, Sándor Békási, Bence Döbrössy, Virág Katalin Bognár, Nóra Radó, Emília Morva, Szabolcs Zsigri, Péter Tari, Edmond Girasek

**Affiliations:** 1 Faculty of Medicine, Institute of Behavioural Sciences, Semmelweis University, Budapest, Hungary; 2 Health Center, Hungarian Charity Service of the Order of Malta, Budapest, Hungary; 3 Telemedicine Workgroup, FitPuli Kft., Győr, Hungary; 4 Regional Directorate, Hungarian Charity Service of the Order of Malta, Budapest, Hungary; 5 Zsigri Háziorvosi Kft., Budapest, Hungary; 6 Ciprus 2007 Kft., Budapest, Hungary; New York City Department of Health and Mental Hygiene, UNITED STATES

## Abstract

**Background:**

With the expansion of digital health, it is imperative to consider intervention techniques in order not to be the cause of even more social health inequalities in underserved populations struggling with chronic diseases. Telemedicine solutions for homeless persons might compensate for shortcomings in access to valuable health services in different settings. The main aim of our research was to examine the attitudes and openness of homeless persons regarding telecare on a Hungarian sample.

**Methods:**

Quantitative survey among homeless people (n = 98) was completed in 4 shelters providing mid- and long-term accommodation in Budapest, Hungary. Attitudes regarding healthcare service accessibility and telecare were measured by a self-developed questionnaire of the research team. Telecare attitude comparison was made with data of a Hungarian weighted reference group of non-homeless persons recruited from 2 primary care units (n = 110).

**Results:**

A significant fraction of homeless people with mid- or long-term residency in homeless shelters did not oppose the use of telecare via live online video consultation and there was no difference compared to the national reference group (averages of 3.09 vs. 3.15, respectively). Results of the homeless group indicate that those more satisfied with healthcare services, in general, manifest more openness to telecare. It is clearly demonstrated by the multivariate analysis that those participants in the homeless group who had problems getting health care in the last year definitely preferred in-person doctor-patient consultations.

**Conclusion:**

Digital health technologies offer a potentially important new pathway for the prevention and treatment of chronic conditions among homeless persons. Based on the attitudes towards telecare, initiating an on-site telecare program for mid- and long-term residents of homeless shelters might enable better care continuity. Our results draw attention to the key factors including building trust in the implementation of such programs among underserved and other vulnerable patient groups.

## Introduction

According to the 2019 report of the European Social Policy Network (ESPN), homelessness had been increasing in the past ten years in most member states of the European Union, including Hungary [1]. The latest report of the European Federation of National Organisations working with the Homeless (FEANTSA) states that at least 700 000 people spend their nights as homeless persons in the European Union [2]. There is no such public database available regarding Hungary. There is a lack of basic demographic studies on the Hungarian homeless population. Little is known about their health status: according to surveys, their self-reported health status is worse compared to that of the general population [3, 4]. Psychiatric disorders, respiratory diseases, hypertension, digestive system diseases, cardiovascular diseases as well as dementia are diagnosed more frequently among them than among the general population [4–8]. According to studies, homeless persons have limited access not only to primary and secondary health care but to a broader spectrum of health-related services, for example, to palliative care, too [9]. The mortality rate of under 65 homeless people may be 4.5–5 times higher than that of the corresponding general population [10].

The geographical separation from health and social care services, problems of accessibility, isolation, stigmatization, discrimination, and individual mental-psychological status may all play an important role in the effectiveness of such programs. Targeted interventions can reduce these burdens [11–13]. The literature attests that the most effective primary care programs use a holistic, multidisciplinary approach, integrating somatic, mental health, and social services. Service provision structures that place homeless patients in the center of attention have a better chance of ensuring continuous care for chronic conditions and lessen the alienation of marginalized people [14].

In Hungary, according to the 2018 survey of the “3rd of February Working Group”, a group of social care professionals, 43% of homeless persons defined themselves as having serious chronic disease and 55% of participants said they were in need of regular medical help [15]. A 2018 study found that the prevalence of chronic illnesses among the Hungarian homeless population was much higher than among the poorest quintile of the general population (79% and 59%, respectively), while homeless persons were much less likely to have had a medical visit in the last 12 months (37.6% and 77.7%) [16].

Digital technology could have an effect on health and wellbeing for the general public. However, limited access to digital technology may contribute to the inaccessibility of services and resources, further disadvantaging a segment of the population that is already marginalized. This very possibility would negatively impact behaviours and stressors, and further contribute to poorer health outcomes for those who are digitally excluded. It needs to be recognized that in the 21^st^ century, digital technology is a health determinant [17, 18].

The COVID-19 pandemic drew vast attention to it, but there were efforts even before that to integrate telemedicine into the health care of homeless persons. In 2019, Calvo et al. conducted a meta-analysis [19] including 50 articles published before 2016 on health information and communications technology (ICT), e-Health, and homelessness. The study looked at the site, frequency, and purpose of digital technology use among homeless populations and the effect it had on their health. The authors concluded that just like among other marginalized groups, the strongest health effects were on mental health, psychological well-being, and stress management. ICT was mostly used for keeping in touch with other individuals, including health care providers, and the use of information technology also shaped their self-identity, making them feel more a part of society.

Shahid et al. [20] undertook diagnostic ophthalmologic examinations using telemedicine in a soup kitchen in an urban U.S. community. Their results emphasized the target population’s multimorbidity and difficulties in accessing health care. Stefancic et al. [21] combined intensive personal case management with telemedicine access in the Pathways Housing First model among homeless persons dealing with severe mental illness in a rural Vermont, USA, environment where a care coordinator and a regional multidisciplinary specialist cooperated in care supervision. In the American ‘Text4Baby’ mHealth program, digital technologies improved adherence among the chronically ill and digital health was an effective supplementary service among homeless persons [22].

A number of studies documented the implementation of telemedicine solutions in social care during the COVID-19 pandemic. In the spring of 2020, a smaller program for homeless persons was launched as a part of the telemedicine program of the Psychiatry Department of the University Salamanca Healthcare Complex [23]. Wood et al. [24] offered telepsychiatry services to homeless adolescents during the pandemic. Heflin et al. [25] reported on their program in the USA where volunteer medical students provided teleconsultations for homeless persons for diagnosis and to maintain care of the chronically ill during the pandemic.

As the literature review supports, providing telemedicine solutions for homeless persons might serve as an efficient, beneficial health service. The main aim of our research was to examine the attitudes and openness of homeless persons regarding telecare services on a Hungarian sample.

This study served also as an assessment tool for analyzing the viability and potential demand of a telecare system planned to be launched by the Hungarian Charity Service of the Order of Malta (HCSOM), a non-governmental organization (NGO) specialized in providing health and social services for homeless persons. Therefore, we were focusing on the attitude towards teleconsultations provided by a physician through live on-line video conference (referred to as telecare) with the help of a care professional (nurse or social worker) on the originating site in an institutional setting.

To the best of our knowledge, the present study is novel in European research analyzing the attitude of homeless persons towards telecare.

## Methods

### Participating homeless establishments

98 homeless persons were recruited into our cross-sectional attitude survey from 4 homeless shelters providing mid- or long-term accommodation in Budapest, Hungary. Based on our previous experience in the health management of homeless persons, we assumed that telecare services are most efficiently linked to such establishments where adequate technological and human resource infrastructure is ensured.

Three of the shelters are operated by the Hungarian Charity Service of the Order of Malta (Budapest, Hungary) and one shelter is operated by a partner institution (Shelter House Foundation, Budapest, Hungary). The selection of the participating homeless shelters in the study was based on one main criterion: the admission of clients to these establishments is determined by the applicant’s health care needs. According to the European Typology of Homelessness and housing exclusion (ETHOS) classification [26], three of the shelters are categorized as 7.2 (supported accommodation), one shelter is categorized as 7.1 (residential care for homeless people).

Only persons having a residential status of 30 days or more in the participating institution were recruited in the study, rough sleepers and persons only using shelters on a temporary or short-term basis were excluded from the study. All participants were of Hungarian nationality. The main characteristics of each participating shelter are summarised in Table 1.

**Table 1:** The main characteristics of the participating shelters.

Institution ID	Type of institution	Gender of accommodated persons	Maximum capacity of the institution	Number of participants of the study	ETHOS classification
**HCSOM 1**	**Shelter for homeless persons**	**Male**	**18 people**	**13 people**	**7.2**
**HCSOM 2**	**Shelter for homeless persons**	**Male**	**40 people**	**23 people**	**7.2**
**HCSOM 3**	**Senior care home for homeless persons**	**Male**	**46 people**	**30 people**	**7.1**
**SHF**	**Shelter for homeless persons**	**Female and Male**	**68 people**	**32 people**	**7.2**

HCSOM: Hungarian Charity Service of the Order of Malta, SHF: Shelter House Foundation.

### Health care services in the participating shelters

Primary care services in all the included establishments are provided by the Health Center of the Hungarian Charity Service of the Order of Malta. Health services cover the full spectrum of primary care, including examining and diagnosing acute illnesses as well as continuous care of chronic conditions of patients. Medication prescribed by the physician is provided free of charge by the Center. Besides primary care, regular physiotherapy, psychiatric care, internal medicine care, addictology consultations, and social case management are also offered in the institutions.

### The process of the survey

Participation in the study was on a voluntary basis. Data collection and analysis of collected surveys were anonymized. Written informed consent statements were obtained in all cases and ethical approval of the study was issued under TUKEB:133/2020 and IV/10927/2020/EKU by the Scientific Research Ethics Committee of the Medical Research Council of Hungary.

To ensure homogeneity, the questionnaire was administered by the same health care assistant or social worker in each given institution. All four interviewers received the same instructions before administering the questionnaires. Influencing respondents, qualifying their responses, or trying to increase response rate were strictly forbidden.

The use of interviewers was due to the respondents’ inexperience in the field. The interviewer was allowed to use an illustrative example to demonstrate the process of telecare if the respondent asked for it in the relevant series of questions but was not permitted to clarify anything regarding the other questions. Data collection took place between 14–21 April, 2020. During this period, the shelters were under lockdown due to the COVID-19 pandemic. Responses were voluntary, response rate in the homeless sample was 56.98%.

### The reference group

As attitudes towards telecare can widely vary from country to country due to the differences in the healthcare systems and the stages of technological development, and no Hungarian data on the general population is available regarding the openness towards telecare, a national reference group was used to provide more context to the results of the index group. Two average-sized and demographic characteristic primary care units were selected as reference groups. These practices were located in Budapest, Hungary, and used the same inclusion criteria as for homeless persons: participants had to have at least one chronic condition managed in the corresponding praxis. Being a homeless person was the only exclusion criterion. All participants were of Hungarian nationality. Questionnaires were administered between 14 April and 31 May, 2020, to 110 respondents.

For comparative analysis, in the case of the reference group, correctional weighting procedure was also executed in order to match it with the homeless group: gender and educational level weights were calculated and used in the following comparative analysis. As a result of the weighting process, the weighted number of respondents in the reference group increased to 112, due to the weight values (there are some respondents who have the largest weight value in more factor). The homeless group was compared with the weighted reference group in each case.

### The structure of the questionnaire

Health care and telecare-related opinions and attitudes were measured by using a questionnaire developed by the research team (see S1 and S2 Files for the Hungarian and English versions of the questionnaire). The questionnaire asked about sociodemographic data (age, gender, level of education, self-defined homelessness, length of being homeless) and frequency of using health services.

The 4.1–4.6. questions were used to inquire about access to health services and opinions about health care (5-grade Likert scale). The following sequence of questions (4.7–4.14.) was used to measure telecare-related attitudes (5-grade Likert scale).

### Statistical analysis

As part of the quantitative analysis, we descriptively examined frequencies, averages, and percentage distributions. Telecare and its various correlates (demographic variables, variables related to access to health services) were compared with single variable analysis using Pearson’s Chi-squared test (χ^2^), with a significance level of p<0.05. When comparing averages, we used the ANOVA model and F-test, with a p <0.05 significance level.

In our multivariate analysis, a binary logistics regression model was executed among the homeless sample. Logistic regression analysis was used to look at the background factors of the statement of *I definitely prefer in-person doctor-patient consultations*. In the multivariate analysis, the dependent variable was the two category variables (not true/completely true) of the above question. The control variables were the following: age, educational status, use of health services, and access to health services in two categories. Independent variables affecting the dependent variables were selected using the enter method in logistic regression. The significance of the regression coefficients of the given variables was described using the p-value of the Wald. Variables with p<0.05 were retained in the final model. The following software was used for the statistical analyses: IBM SPSS 27.0 (IBM Corporation, Armonk, NY, USA).

## Results

### Demographic data

98 homeless persons participated in the study. The weighted reference group consisted of 112 people. 65.3% of the homeless group defined themselves as homeless while 34.7% did not. Male respondents made up 90.8% of the homeless group and 92.0% of the weighted reference group. The age difference between the two groups was not significant (mean age was 64.0 for the homeless group and 63.1 for the reference group). Key demographic parameters of the three groups (homeless, unweighted, and weighted reference group) are shown in Table 2.

**Table 2:** Main demographic characteristics of the index and the reference group.

		Homeless persons		Reference population		Reference population (weighted)	
Number of respondents		98		110		112	
		n	%	n	%	n	%
Gender	Male	89	90.8%	61	55.5%	103	92.0%
	Female	9	9.2%	49	44.5%	9	8.0%
	Total	98	100.0%	110	100.0%	112	100.0%
Do you consider yourself to be a homeless person?	No	33	34.7%	N / A		N / A	
	Yes	62	65.3%				
	Total	95	100.0%				
How many years have you been homeless?	0–5	25	41.0%	N / A		N / A	
	6–10	10	16.4%				
	11–15	10	16.4%				
	16 or more	16	26.2%				
	Total	61	100.0%				
What is your highest level of education?	Less than 8 years of elementary school	3	3.1%	3	2.7%	4	3.6%
	Elementary school	25	25.8%	6	5.5%	19	17.0%
	Vocational school	48	49.5%	25	22.7%	69	61.6%
	High school	15	15.5%	29	26.4%	14	12.5%
	Higher education (college or university)	6	6.2%	47	42.7%	6	5.4%
	Total	91	100.0%	100	100.0%	112	100.0%
How often do you see a doctor/use health care services? *	Semi-annually or more frequently	64	67.40%	98	91.60%	96	88.9%
	Annually or less frequently	31	32.60%	9	8.40%	12	11.1%
	Total	95	100.0%	107	100.0%	112	100.0%
Age		mean	n	mean	n	mean	n
	Mean (p < 0.05)	64.0	98	63.1	110	112	62.9

The cells marked with

* represent p < 0.05 between the homeless and the weighted reference group.

As one of the correctional weights was education level, there was no significant difference between the homeless and adjusted reference group. Among the homeless group, the biggest proportion consisted of those who became homeless between 2016–2020 (39.6%). 20.0% of the group became homeless between 2010–2015, while 16.9% of the group had been homeless for over 10 years and 23.8% for over 15.

67.5% of the homeless group utilized health services every six months or more frequently and 32.6% less than annually. In the reference group, 88.9% used healthcare services every half year or less and only 11.14% less than annually (p < 0.001).

### Telecare-related attitudes

First, we compared the health care access and telecare-related attitudes of the two groups along scale averages. Significant differences were observed for the following 3 statements:

- *In the last year*, *I only saw a doctor when I had acute complaints* (more characteristic of the homeless group).
- *It is important to have a live video consultation with such a doctor whom I met previously in person* (less characteristic of the homeless group).
- *Having a live video consultation with a doctor might improve my health status* (less characteristic of the homeless group).

There was no significant difference in the willingness to try a live video consultation (averages of 3.09 vs. 3.15) (Table 3).

**Table 3:** Means of attitude responses by the homeless persons and the reference group.

	Homeless persons		Reference (weighted)	
	Mean	Std. Deviation	Mean	Std. Deviation
In the last year, I could take my prescribed medication regularly.	4.46	1.08	4.49	0.93
I feel my chronic conditions are managed adequately.	4.36	1.21	4.10	1.10
In the last year, I only saw a doctor when I had acute complaints.	4.03	1.42	2.80	1.41
I feel I have to wait a long time to receive health care in Hungary.	2.91	1.62	2.89	1.21
In the last year, I had problems with getting adequate health care.	1.98	1.44	1.82	0.98
In the last year, I sometimes felt I am not dealt with well in the health care setting.	1.96	1.51	1.70	0.90
I would gladly try discussing my chronic condition with a doctor through a live video consultation.	3.09	1.74	3.15	1.58
It would help if I could have a video consultation with a doctor at a pre-arranged appointment.	3.14	1.79	3.05	1.49
I would have trust in a doctor in a live video consultation.	3.24	1.77	3.53	1.32
It is important to have a live video consultation with such a doctor whom I met previously in person.	3.14	1.71	3.78	1.46
Having a live video consultation with a doctor might improve my health status.	2.20	1.39	2.71	1.14
I definitely prefer in-person doctor-patient consultations.	4.06	1.25	3.75	1.36
I don’t feel that talking to a doctor through a live video consultation is safe.	2.94	1.66	2.65	1.44
I would feel uncomfortable talking to a doctor through a live video consultation.	2.53	1.71	2.77	1.58

The cells marked with grey show p < 0.05.

Next, we merged the answer categories of the Likert scale 1–3 into ‘rather not true’ and answers 4–5 into ‘rather true’. During cross tabulation analysis, the following variables manifested significant differences.

The homeless group was significantly more likely to seek medical attention in case of acute complaints only (p<0.001). Having problems getting adequate care in the last year was significantly more characteristic of the homeless group (p<0.022). Feeling that they are not treated adequately in the healthcare system was more characteristic of the homeless group, too (p<0.001).

**Fig 1:**
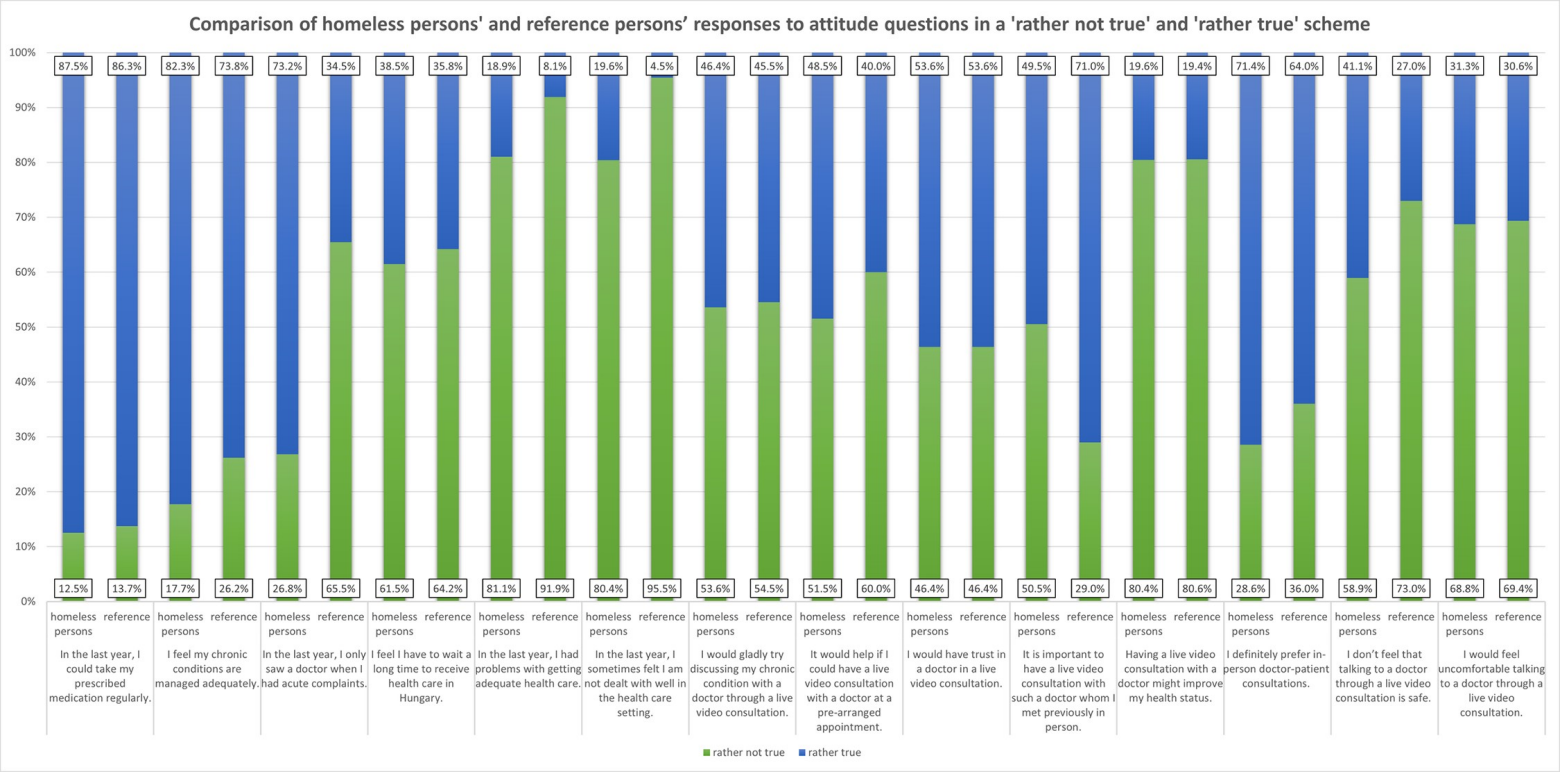
Comparison of homeless persons’ and reference persons’ responses to attitude questions in a ’rather not true’ and ’rather true’ scheme.

In case of the telecare statements, knowing the doctor in person with whom they were having a teleconsultation was significantly less important for them (p<0.002) and they also felt that telecare consultations were less secure (p<0.033) (Fig 1).

### Factors associated with positive or negative telecare attitudes

We also examined the factors associated with positive or negative telecare attitudes within the homeless group. First, we looked at the demographic variables regarding the telecare questions. No significant association was found between the telecare statements and gender, level of education or self-defined homeless status.

For the following 3 questions, significantly less mean aged respondents manifested significantly higher agreement:

- *I would gladly try discussing my chronic condition with a doctor through a live video consultation*.
- *It would help if I could have a live video consultation with a doctor at a pre-arranged appointment*.
- *I would have trust in a doctor in a live video consultation*.

People who were homeless for a longer period expressed more agreement with the statement *I would feel uncomfortable talking to a doctor through a live video consultation* (Table 4).

**Table 4:** Association between age, years of homelessness and telecare attitude within the group of homeless persons.

	rather not true	rather true	rather not true	rather true
	Age (mean, years)		How many years you have been homeless? (mean, years)	
I would gladly try discussing my chronic condition with a doctor through a live video consultation.	66.69	60.98	12.86	10.03
It would help if I could have a live video consultation with a doctor at a pre-arranged appointment.	66.62	61.30	14.11	8.94
I would have trust in a doctor in a live video consultation.	65.98	62.37	14.73	8.76
It is important to have a live video consultation with such a doctor whom I met previously in person.	65.39	62.67	11.87	10.79
Having a live video consultation with a doctor might improve my health status.	64.50	61.78	11.50	9.00
I definitely prefer in-person doctor-patient consultations.	63.61	64.16	8.83	12.28
I don’t feel that talking to a doctor through a live video consultation is safe.	62.96	65.77	11.14	11.70
I would feel uncomfortable talking to a doctor through a live video consultation.	63.05	66.70	9.17	16.05

The cells marked with grey represent p < 0.05.

In the homeless group, we looked at the association of telecare attitudes and frequency of use and access to health services. There was a tendency (p<0.087) that people who had problems taking prescribed medication in the last year were less willing to try telecare. Having trust in a doctor in a live video consultation was significantly less characteristic of homeless persons who said they had problems taking regularly prescribed medication in the past year (p<0.028). It could be also observed that those homeless persons who reported problems taking regularly prescribed medication in the past year would have definitely preferred in-person doctor-patient consultations (p< 0.033).

We also found that preferring in-person doctor-patient consultations was significantly more characteristic of those reporting problems with getting adequate health services in the past year (p<0.048). They also didn’t feel that talking to a doctor through a live video consultation was safe (p<0.018). Video consultations were also more uncomfortable for those reporting problems with getting adequate health services in the past year (p<0.011). It was significantly less important to have a video consultation with such a doctor whom they met previously in person for those who used health care services annually or less frequently (p<0.001). Those who felt they hadn’t been dealt with properly in a health care setting in the last year expressed the opinion that telecare was less safe (p<0.05) (Table 5).

**Table 5:** Association of telecare attitudes with frequency of use and access to health services.

		I would gladly try discussing my chronic condition with a doctor through a live video consultation.		It would help if I could have a live video consultation with a doctor at a pre-arranged appointment.		I would have trust in a doctor in a live video consultation.		It is important to have a live video consultation with such a doctor whom I met previously in person.		Having a live video consultation with a doctor might improve my health status.		I definitely prefer in-person doctor-patient consultations.		I don’t feel that talking to a doctor through a live video consultation is safe.		I would feel uncomfortable talking to a doctor through a live video consultation.	
		rather not true	rather true	rather not true	rather true	rather not true	rather true	rather not true	rather true	rather not true	rather true	rather not true	rather true	rather not true	rather true	rather not true	rather true
How often do you see a doctor/use health care services?	semi-annually or more frequently	33	30	32	31	28	35	24	39	47	11	18	46	33	28	40	23
		52.4%	47.6%	50.8%	49.2%	44.4%	55.6%	38.1%	61.9%	81.0%	19.0%	28.1%	71.9%	54.1%	45.9%	63.5%	36.5%
	annually or less frequently	17	14	17	14	16	15	24	7	25	6	10	21	21	10	24	7
		54.8%	45.2%	54.8%	45.2%	51.6%	48.4%	77.4%	22.6%	80.6%	19.4%	32.3%	67.7%	67.7%	32.3%	77.4%	22.6%
In the last year, I could take my prescribed medication regularly.	rather not true	9	3	8	4	9	3	5	7	7	3	0	12	3	8	6	5
		75.0%	25.0%	66.7%	33.3%	75.0%	25.0%	41.7%	58.3%	70.0%	30.0%	0.0%	100.0%	27.3%	72.7%	54.5%	45.5%
	rather true	41	42	40	43	34	49	42	41	65	15	28	56	53	29	59	24
		49.4%	50.6%	48.2%	51.8%	41.0%	59.0%	50.6%	49.4%	81.3%	18.8%	33.3%	66.7%	64.6%	35.4%	71.1%	28.9%
I feel my chronic conditions are managed adequately.	rather not true	10	7	9	8	10	7	9	8	12	2	4	13	7	9	9	8
		58.8%	41.2%	52.9%	47.1%	58.8%	41.2%	52.9%	47.1%	85.7%	14.3%	23.5%	76.5%	43.8%	56.3%	52.9%	47.1%
	rather true	40	38	39	39	33	45	38	40	60	16	24	55	49	28	55	22
		51.3%	48.7%	50.0%	50.0%	42.3%	57.7%	48.7%	51.3%	78.9%	21.1%	30.4%	69.6%	63.6%	36.4%	71.4%	28.6%
In the last year, I only saw a doctor when I had acute complaints.	rather not true	17	9	14	12	15	11	14	12	19	4	6	20	16	9	16	10
		65.4%	34.6%	53.8%	46.2%	57.7%	42.3%	53.8%	46.2%	82.6%	17.4%	23.1%	76.9%	64.0%	36.0%	61.5%	38.5%
	rather true	35	36	36	35	30	41	35	36	55	14	22	49	40	30	50	20
		49.3%	50.7%	50.7%	49.3%	42.3%	57.7%	49.3%	50.7%	79.7%	20.3%	31.0%	69.0%	57.1%	42.9%	71.4%	28.6%
I feel I have to wait a long time to receive health care in Hungary.	rather not true	31	28	30	29	28	31	33	26	45	12	19	40	37	21	42	17
		52.5%	47.5%	50.8%	49.2%	47.5%	52.5%	55.9%	44.1%	78.9%	21.1%	32.2%	67.8%	63.8%	36.2%	71.2%	28.8%
	rather true	20	17	19	18	16	21	15	22	28	6	9	28	19	17	23	13
		54.1%	45.9%	51.4%	48.6%	43.2%	56.8%	40.5%	59.5%	82.4%	17.6%	24.3%	75.7%	52.8%	47.2%	63.9%	36.1%
In the last year, I had problems with getting adequate health care.	rather not true	42	35	39	38	36	41	42	35	63	13	26	51	48	27	57	20
		54.5%	45.5%	50.6%	49.4%	46.8%	53.2%	54.5%	45.5%	82.9%	17.1%	33.8%	66.2%	64.0%	36.0%	74.0%	26.0%
	rather true	10	8	11	7	9	9	7	11	10	4	2	16	6	12	7	10
		55.6%	44.4%	61.1%	38.9%	50.0%	50.0%	38.9%	61.1%	71.4%	28.6%	11.1%	88.9%	33.3%	66.7%	41.2%	58.8%
In the last year, I sometimes felt I am not dealt with well in the health care setting.	rather not true	42	36	41	37	37	41	42	36	62	14	23	55	49	28	56	22
		53.8%	46.2%	52.6%	47.4%	47.4%	52.6%	53.8%	46.2%	81.6%	18.4%	29.5%	70.5%	63.6%	36.4%	71.8%	28.2%
	rather true	10	9	9	10	8	11	7	12	12	4	5	14	7	11	10	8
		52.6%	47.4%	47.4%	52.6%	42.1%	57.9%	36.8%	63.2%	75.0%	25.0%	26.3%	73.7%	38.9%	61.1%	55.6%	44.4%

The cells marked with grey show significant interrelation (p < 0.05).

Finally, we used a logistic regression model to see which variables were associated with the *I definitely prefer in-person doctor-patient consultations* statement among the homeless sample. Our dependent variable was our 2 category variables (rather true/rather not true) of the question above. Age (as a continuous variable), level of education (5 categories), frequency of use of health services (every six months or more frequently, rarer than a year) as well as the two category variables of the 4.1–4.6. questions were entered in the model.

The logistic regression model was significant (Nagelkerke R square is 0.292). The independent variables used in the model explain a significant part of the heterogeneity of the dependent variable. The R square shows the proportion of the heterogeneity explained by the variables used in the model. Besides the independent variables, preferring in-person doctor-patient consultations was significantly associated with the frequency of use of health services and with the following statement *In the last year*, *I had problems with getting adequate health care* (OR = 10.684, CI:1.108–102.999) (Table 6).

**Table 6:** The logistic regression explanation model of the *I definitely prefer in-person doctor-patient consultations* variable.

Nagelkerke R-square = 0,296	B	S.E.	Wald	df	Sig.	Odds Ratio (OR)	95% C.I.for EXP(B)	
							Lower	Upper
Age in years	0.015	0.032	0,226	1	0.634	1.015	0.954	1.080
Level of education—reference: less than 8 years of elementary school			2.110	4	0.716			
Level of education (1) elementary school	-20.084	20562.316	0.000	1	0.999	0.000	0.000	
Level of education (2) vocational school	-19.202	20562.316	0.000	1	0.999	0.000	0.000	
Level of education (3) secondary school degree	-19.540	20562.316	0.000	1	0.999	0.000	0.000	
Level of education (4) higher education (college or university)	-20.217	20562.316	0.000	1	0.999	0.000	0.000	
How often do you see a doctor/ use healthcare facilities?	-0.154	0.228	0.458	1	0.498	0.857	0.549	1.339
In the last year, I could take my prescribed medication regularly. (0 = rather not true; 1 = rather true)	-21.280	11015.379	0.000	1	0.998	0.000	0.000	
I feel my chronic conditions are managed adequately. (0 = rather not true; 1 = rather true)	0.898	0.995	0.815	1	0.367	2.455	0.349	17.241
In the last year, I only saw a doctor when I had acute complaints. (0 = rather not true; 1 = rather true)	-0.575	0.614	0.879	1	0.349	0.563	0.169	1.873
I feel I have to wait a long time to receive health care in Hungary. (0 = rather not true; 1 = rather true)	0.035	0.633	0.003	1	0.956	1.035	0.299	3.582
In the last year, I had problems with getting adequate health care. (0 = rather not true; 1 = rather true)	2.369	1.156	4.198	1	0.040	10.684	1.108	102.999
In the last year, I sometimes felt I am not dealt with well in the health care setting. (0 = rather not true; 1 = rather true)	-1.046	1.006	1.081	1	0.298	0.351	0.049	2.524
Constant	40.321	23326.962	0.000	1	0.999	324379789920998000		

Dependent variable: *I definitely prefer in-person doctor-patient consultations* (0 = rather not true, 1 = rather true).

## Discussion

The COVID-19 pandemic has rapidly hastened the spread of telemedicine solutions all over the world. It must be taken into consideration whether these digital health initiatives provide novel care options for underserved populations regarding better access to healthcare services. Our results are congruent with the literature demonstrating significant differences in access to and use of health services within the homeless population: this group is less inclined to feel that they have access to adequate services. It is also important to study the attitudes, literacy, and feasibility opportunities this population has with regard to telemedicine.

The results about telecare attitudes indicate that a significant fraction of homeless people with mid- or long-term residency in homeless shelters do not oppose the use of telecare via live online video consultation and there is no difference compared to the national reference group. At the same time, the homeless group is more likely to favour in-person doctor-patient consultations. They are also less inclined to feel that telecare is safe as compared to the reference group. Results of the homeless group indicate that those more satisfied with health care services, in general, manifest more openness to telecare. Although these results support a general openness of this population, a subpopulation of homeless persons reported struggling with getting access to health care services is more likely to favour in-person doctor-patient consultations.

It is clearly demonstrated by the multivariate analysis that those participants in the homeless group who had problems getting health care in the last year definitely preferred in-person doctor-patient consultations. The results of the bivariate analysis also indicate that problems with taking prescribed medication, inadequate management of chronic conditions, long waiting times, and the feeling of not being dealt with well in health care all point to a greater disassociation from telecare.

The observation could be made that younger participants were more open to telemedicine. The systematic review of Calvo et al. supports this evidence when they note that young homeless persons used digital tools very much like their contemporaries, especially when it comes to social media [27].

The cross-sectional study of Reed et al. [28] looked at data from 1.1 million patients with 2.2 million primary care visits and concluded that patients from neighbourhoods characterized by lower socioeconomic status were significantly less willing to participate in live video visits. Telemedicine choice was significantly associated with access to technology. Patients whose insurance plans mandated higher co-payment for in-person consultations had a higher tendency to choose a telemedicine visit than patients with lower cost-sharing. Similarly, patients with longer relative driving time from home to the medical facility were significantly more likely to choose a telemedicine visit. Patients were also more likely to schedule a telemedicine visit if they were visiting their own primary care physician than visiting another primary care physician.

Based on our results, we may assume that in telecare, considerable trust is required between the service provider and the recipient. Trust is a pillar of the doctor-patient relationship, it affects the possibilities of access to care, is essential to adherence, compliance, and patient satisfaction, and improves clinical outcomes [29]. People who feel they receive adequate care have a bigger chance of having trust in telecare, too. In this population, ‘digital trust’ is not primarily related to information privacy and security concerns or having faith in technology. Here, trust is more related to having access to quality care and conditions necessary for the continuity of care [30].

Trust in the field of digital health is a key question both on the provider and receiver side. The World Health Organization (WHO) Global Strategy on Digital Health recognizes that building trust in digital health is vital for the overall success of these solutions [31]. Adjekum, Blasimme, and Vayena [29] reviewed 278 qualitative, quantitative, mixed-methods, and intervention studies dealing with trust and digital health published in 40 different countries between 1998 and 2017, of whom 51 studies researched trust and telemedicine specifically. What they found was that patients’ and healthcare providers’ trust in digital health depended on a complex interplay of facilitators and barriers. Altruism, fair data access, ease of use, self-efficacy, sociodemographic factors, recommendation by other users, usefulness, customizable design features, interoperability, privacy, initial face-to-face contact, guidelines for standardized use, stakeholder engagement, improved communication, decreased workloads, and service provider reputation enabled trust-building. In contrast, the negative factors were excessive costs, limited accessibility, fear of data exploitation, insufficient training, defective technology, poor information quality, inadequate publicity and time-consuming service.

Regarding homeless individuals, our trust-related results support what has been established by the systematic review of Parker et al [32]. This review concludes that general unease and mistrust were barriers inhibiting digital health technology use by vulnerable patients. In their study, Gabrielian et al. noted that according to staff reports, homeless veterans expressed suspicions about in-home digital technology and health monitoring devices [33].

Research involving homeless persons is usually done on small, non-representative samples, and this is true for our samples as well [34]. A further limitation of the present study is that we did not attempt to differentiate among the subpopulations of homeless persons and excluded homeless persons who were rough sleepers or used shelters on a short-term basis. Based on the Calvo et al. systematic review [19], there are well-identified subpopulations of homeless persons such as pregnant women, the youth, the elderly, mentally challenged people, and people afflicted with severe addictions whose telecare attitudes might differ from the one of the participating populations. Another limitation is the differences in gender and levels of education between the index and reference groups, this difference was administered by using correctional weights on the reference group.

Furthermore, we did not assess the digital technology skills of the respondents (i.e., their digital literacy) and their access to such devices or platforms. For broader research on the telemedical opportunities among homeless persons such as mobile health or remote monitoring, a survey including technological aspects should be undertaken as well.

## Conclusion

The results of our quantitative exploratory attitude survey support the notion that implementing telecare programs among homeless persons in need of regular medical care is a worthy endeavour. In a model, where health and social care activities form a holistic service portfolio, on-site digital health services might serve as an additional tool in homeless shelters.

Trust in a telemedicine service as a major facilitator can primarily be achieved gradually in health screening and long-term management of chronic conditions. Therefore, this is the form of digital health service we recommend implementing foremost among homeless persons. Because of the associated uncertainty, telemedicine solutions for acute and emergency patient care should be considered only as a further development of a consistently functioning service. This requires a system that is already operational where all the participants are familiar with the structure and its use is routine for them.

As digital health can be a barrier for populations with no or very limited access to mobile devices and digital platforms, telemedicine services can most effectively and easily be offered if they are organized through the shelters as the necessary basic infrastructure and technology are already available in most of these establishments. The telemedicine support team, ideally consisting of members of the core care team, must be present on the originating site so they are able to manage uncertainties and troubleshoot possible technical difficulties. This can lead to better user experience, adherence, and health service quality.

## Supporting information

S1 File Telecare attitude questionnaire in Hungarian. (PDF)

S2 File Telecare attitude questionnaire in English. (PDF)
