# Supplementary material for: Exploratory attitude survey of homeless persons regarding telecare services in shelters providing mid- and long-term accommodation: The importance of trust
Source: PLoS One. 2022 Jan 6;17(1):e0261145. doi: 10.1371/journal.pone.0261145 (PMC8735598; doi:10.1371/journal.pone.0261145)
Supplement: S1 File — (PDF) [file pone.0261145.s001.pdf]

Magyar Máltai Szeretetszolgálat Telemedicina kérdőív

Név (Monogram): **Születési idő:** **Nem:** Nő / Férfi

1. Hajléktalannak tartja-e magát? NEM IGEN (ha igen, mióta?) ÉV:

2. Mi a legmagasabb iskolai végzettsége?

8 általános alatt 8 általános szakmunkás érettségi főiskola, egyetem nem válaszolok

3. Milyen gyakran jár orvoshoz/ vesz igénybe egészségügyi ellátást?

Havonta többször 1-2 havonta Félévente Évente Ritkábban, mint egy év

4. Az alábbi kérdéseknél x-szel jelöljük az alany válaszát.

KÉRDÉS	VÁLASZ				
	Nem igaz	Kevéssé igaz	Valamennyire igaz	Többnyire igaz	Teljesen igaz
Rendszeresen szedett gyógyszereimet az elmúlt 1 évben mindig be tudtam venni.					
Úgy érzem, krónikus betegségeim megfelelően karban van tartva.					
Az elmúlt 1 évben csak akkor fordultam orvoshoz, ha akut panaszom volt.					
Úgy érzem, a hazai egészségügyben sokat kell várnom arra, hogy ellátást kapjak.					
Az elmúlt 1 évben előfordult, hogy gondot okozott, hogy megfelelő ellátáshoz jussak.					
Az elmúlt 1 évben előfordult, hogy úgy éreztem, nem megfelelően viszonyultak hozzám az egészségügyi ellátórendszerben.					

KÉRDÉS	VÁLASZ				
	Nem igaz	Kevéssé igaz	Valamennyire igaz	Többnyire igaz	Teljesen igaz
Szívesen kipróbálnám, hogy krónikus betegségemet számítógépes elő videókapcsolaton keresztül beszéljem meg egy orvossal.					
Segítene, ha egy előre megbeszélt időpontban, várakozás nélkül tudnám igénybe venni a videón keresztüli orvosi beszélgetést.					
Bizalommal fordulnék élő, videókapcsolatban levő orvoshoz.					
Fontos, hogy olyan orvossal beszéljek a videón keresztül, akivel korábban személyesen is találkoztam.					
Lehet, hogy javulna az egészségi állapotom, ha élő videón keresztül beszélgetnék egy orvossal.					
Mindenképpen a személyes orvos-beteg találkozást részesítem előnyben.					
Nem érzem biztonságosnak, ha élő videókapcsolaton keresztül beszélgetnék egy orvossal.					
Kényelmetlenül érezném magam, ha élő videókapcsolaton beszélgetnék egy orvossal.					
