# Supplementary material for: Exploratory attitude survey of homeless persons regarding telecare services in shelters providing mid- and long-term accommodation: The importance of trust
Source: PLoS One. 2022 Jan 6;17(1):e0261145. doi: 10.1371/journal.pone.0261145 (PMC8735598; doi:10.1371/journal.pone.0261145)
Supplement: S2 File — (PDF) [file pone.0261145.s002.pdf]

Telemedicine Questionnaire by the Hungarian Charity Service of the Order of Malta

Name (initials): **Date of Birth:** **Gender:** Female/Male

1. Do you consider yourself to be a homeless person? No Yes **(If yes, since when?) Year:**

2. What is your highest level of education?

Less than 8 years of elementary school Elementary school Vocational school
Secondary school degree College/university degree I'd rather not answer

3. How often do you see a doctor/use health care services?

A few times each month Every 1-2 months Every 6 months Every year Less than every year

4. Please mark your answer with an x in the appropriate box.

Questions	Answer				
	not true	just slightly true	somewhat true	mostly true	completely true
4.1. In the last year, I could take my prescribed medication regularly.					
4.2. I feel my chronic conditions are managed adequately.					
4.3. In the last year, I only saw a doctor when I had acute complaints.					
4.4. I feel I have to wait a long time to receive health care in Hungary.					
4.5. In the last year, I had problems with getting adequate health care.					
4.6. In the last year, I sometimes felt I am not dealt with well in the health care setting.					

Questions	Answer				
	not true	just slightly true	somewhat true	mostly true	completely true
4.7. I would gladly try discussing my chronic condition with a doctor through a live video consultation.					
4.8. It would help if I could have a live video consultation with a doctor at a pre-arranged appointment.					
4.9. I would have trust in a doctor in a live video consultation.					
4.10. It is important to have a live video consultation with such a doctor whom I met previously in person.					
4.11. Having a live video consultation with a doctor might improve my health status.					
4.12. I definitely prefer in-person doctor-patient consultations.					
4.13. I don't feel that talking to a doctor through a live video consultation is safe.					
4.14. I would feel uncomfortable talking to a doctor through a live video consultation.					
